# Taming the Notch Transcriptional Regulator for Cancer Therapy

**DOI:** 10.3390/molecules23020431

**Published:** 2018-02-15

**Authors:** Luca Tamagnone, Serena Zacchigna, Michael Rehman

**Affiliations:** 1 Laboratory of Cancer Cell Biology, Candiolo Cancer Institute-FPO, IRCCS, Str. Prov. 142, 10060 Candiolo, TO, Italy; 2 Department of Medical, Surgical and Health Sciences, University of Trieste, 34127 Trieste, Italy; zacchign@icgeb.org; 3 Department of Oncology, University of Torino, c/o IRCCS, S.P. 142, 10060 Candiolo, TO, Italy; 4 Cardiovascular Biology Laboratory, International Centre for Genetic Engineering and Biotechnology (ICGEB), 34149 Trieste, Italy

**Keywords:** Notch transcription complex, gamma secretase inhibitors, combination therapy, blocking antibody, clinical trials, combination therapy

## Abstract

Notch signaling is a highly conserved pathway in all metazoans, which is deeply involved in the regulation of cell fate and differentiation, proliferation and migration during development. Research in the last decades has shown that the various components of the Notch signaling cascade are either upregulated or activated in human cancers. Therefore, its downregulation stands as a promising and powerful strategy for cancer therapy. Here, we discuss the recent advances in the development of small molecule inhibitors, blocking antibodies and oligonucleotides that hinder Notch activity, and their outcome in clinical trials. Although Notch was initially identified as an oncogene, later studies showed that it can also act as a tumor suppressor in certain contexts. Further complexity is added by the existence of numerous Notch family members, which exert different activities and can be differentially targeted by inhibitors, potentially accounting for contradictory data on their therapeutic efficacy. Notably, recent evidence supports the rationale for combinatorial treatments including Notch inhibitors, which appear to be more effective than single agents in fighting cancer.

## 1. Diversity of Notch Signaling Mechanisms

This is the centennial of the discovery of the Notch gene, which was first identified by Thomas H. Morgan and colleagues in 1917 in fruit flies, where spontaneous mutations in a specific locus of the X chromosome produced notches at the wing margin [1,2]. Molecular genetic studies followed, but it took until the 1980s to accomplish the actual cloning of *Drosophila Notch* gene by Artavanis-Tsakonas, Young, and colleagues [3,4]. Notably, the Notch pathway is highly conserved from sea urchins to humans. In contrast to flies that have only one gene, there are four Notch receptors in mammals, Notch1 to Notch4. Notch signaling regulates differentiation, proliferation, apoptosis, migration, and angiogenesis, as well as stem cell growth and survival during development and disease [5,6,7,8,9].

Notch proteins are single pass transmembrane heterodimeric receptors. They are synthesized as single-chain precursors that undergo processing and modification in the endoplasmic reticulum and the Golgi apparatus to produce the mature forms [10]. In particular, Notch precursor undergoes a first proteolytic cleavage (S1) in the Golgi to form a heterodimer, which represents the mature form of the receptor. This heterodimer sits on the plasma membrane and is composed of a large extracellular domain (N-ECD) and a membrane-tethered intracellular domain (N-ICD). Notch receptors are activated by ligands of the Delta-like (Dll) and Jagged (Jag) families that are exposed on the surface of adjacent cells, thereby binding the N-ECD *in trans* and acting in a juxtracrine manner. In total, there are five Notch ligands that belong to Delta like ligand (DLL) and Jagged families—Dll1, Dll3, Dll4, Jag1, and Jag2. Dll and Jagged ligands contain Delta-Serrate-Lag2 (DSL) domain and EGF (Epidermal Growth Factor) repeats that interact with EGF-like repeats found in the Notch ectodomain to activate the signaling cascade. Interactions between Notch and its ligands *in cis*, instead, are reported to be inhibitory [11]. The glycosylation of EGF repeats is an important modulator of Dll vs Jagged signaling; for instance, Fringe glycosyltransferases (that add GlcNAc to O-fucose on Notch EGF repeats) can enhance the binding of Dll ligands, compared to Jagged, to the Notch receptor [12]. Upon ligand binding, Notch is sequentially cleaved at the plasma membrane by two other proteases. Extracellular S2 cleavage is due to the tumor necrosis factor-alpha converting enzyme (TACE), whereas S3 intramembrane cleavage is operated by a gamma secretase, which catalyzes the release of the intracellular domain of Notch (N-ICD) into the cytoplasm. N-ICD then translocates inside the nucleus and forms a complex with RBPjk/CSL (CBF1/Su(H)/Lag-1) [13]. RBPjk is a DNA-binding transcription factor, which acts in a large complex with other proteins. It is the prime effector of Notch-associated functions. Normally RBPjk acts as a repressor of Notch transcriptional target genes due to its association with SMRT corepressor, CIR, and histone deacetylase-1 (HDAC1). N-ICD binding disrupts this inhibitory complex, releasing the repressor proteins and fostering the formation of a new complex of RBPjk with SKIP, MAML1, PCAF, and GCN5, which is able to induce gene expression. Notch signaling regulates a diverse set of genes that impinge on several cellular functions [6]. Among the known Notch-RBPjk targets are the genes belonging to the HES and HEY families, NF-κB1, NF-κB2, STAT6, p27/Kip1, ErbB2, CyclinD1, cMyc, p21. 

DSL-ligand- and RBPjk- independent Notch-initiated signaling has also been reported, referred to as “non-canonical” Notch pathway [14,15]. While the canonical pathway plays a major and well defined role in stem cell maintenance and cell fate determination during development, and it regulates several cancer hallmarks in tumorigenesis, the functions of the non-canonical pathway are poorly characterized [14]. Neither the targets nor the mediators of the non-canonical pathway are clearly defined; however, N-ICD has been reported to associate with other transcription factors distinct from RBPjk, such as SMAD3, YY1, HIF1α, and NF-κB [16,17,18,19]. In one study, it was shown that IL-6, an important pro-inflammatory cytokine in tumors, was upregulated by Notch1-ICD in breast cancer cells with p53 mutation or deficiency, independent of RBPjk [20]; IKKα and IKKβ were implicated in this mechanism, but whether they can form a complex with N-ICD needs to be elucidated. Several studies illustrated the crosstalk between hypoxia-induced HIF and Notch transcription factors; for instance, HIF1α was found to interact and stabilize the N-ICD, leading to enhancement of the canonical Notch pathway [21,22,23]. On the other hand, it was shown that the negative regulator FIH-1 (Factor Inhibiting HIF1) can bind and functionally inactivate N-ICD by hydroxylation [18]. In addition, RBPjk–independent Notch signaling was reported to activate the PI3K pathway in cervical cancer cell lines, implicating the activity of Deltex1, an E3-ubiquitin ligase [24].

## 2. Diversity of Notch Signaling Activities in Cancer

Ellisen and coworkers were the first to associate Notch with cancer; they reported that in T-cell acute lymphocytic leukemia (T-ALL), Notch1 gets constitutively activated due to chromosomal translocation t(7;9)(q34; q34.3), found in about 1% of T-ALL patients [21]. Later studies showed that more than 50% of T-ALLs actually show activating Notch1 mutations in the heterodimerization (HD) and ‘proline, glutamic acid, serine, threonine-rich’ (PEST) domains [22]. Remarkably, the majority of solid human cancers bear deregulated expression of Notch pathway genes, rather than genetic changes [23]. Notch1 is the best-studied member of the family, and it is often overexpressed in human breast, colorectal, lung, pancreas, and prostate cancers [6]. Notch1 expression is induced by hypoxia, which is a common event in growing tumors [24,25]. Moreover, Notch signaling is activated downstream of a range of pathways deregulated in cancer, such as cMyc, p53, PI3K, or RAS [26,27,28,29]. Several miRNAs can furthermore control the Notch pathway, e.g., miR34a and miR326 have been shown to target both Notch1 and Notch2 in gliomas and pancreatic cancer [30,31,32], and Notch3 3′ UTR can be targeted by miR206 [33]. 

Notch activity regulates several cancer hallmarks, such as cancer cell survival, proliferation, migration, invasion, and metastasis, as it transcriptionally modulates a range of signaling pathways. For instance, the ERK pathway and cyclinD1 seem to mediate Notch1-dependent control of cell survival and proliferation [34,35,36]. Moreover, Notch regulates mTOR/Akt and NF-κB pathways, eventually inhibiting apoptosis in breast cancer cells [37,38,39]. Notch1 and its ligands were found to be overexpressed in prostate cancer compared to normal tissue [40]. Notably, prostate cancers are commonly characterized by inactivation of the tumor suppressor PTEN [41,42], which is negatively regulated by Hes1, a well-known effector of canonical Notch signaling [43]. In addition, Kwon and coworkers showed that in PTEN null mice Notch signaling was required for metastatic dissemination, through the induction of epithelial to mesenchymal transition (EMT) in a FOXC2-dependent manner [40]. Indeed, loss of E-cadherin and EMT are key events in prostate cancer metastasis [44]. EMT is characterized by increased expression of a series of transcription factors (including Snail, Slug, Twist, Zeb1, and Zeb2), and the Notch pathway induces their expression in prostate as well as in breast, pancreatic, and colon cancers [45,46,47]. Notably, in prostate cancer cells, Notch was also shown to regulate an EMT-like phenotype through the upregulation of the semaphorin receptor PlexinD1, impinging on SLUG [48]. Moreover, PlexinD1 expression correlates with Notch1 and Notch3 in different cancer types [48], and its role in metastatic tumor progression has been further demonstrated in colon cancer, melanoma, and ovarian cancer [49,50]. In apparent contradiction with these data, in endothelial cells Notch appears to negatively regulate PlexinD1 expression [51]. Thus, the Notch-PlexinD1 axis seems to exert a range of effects in diverse cells, warranting further studies to fully define its role in cancer. 

Notably, in certain contexts, Notch signaling has been shown to be tumor suppressive, such as in head and neck carcinomas, and in pancreatic cancer [52,53]. In squamous carcinomas of skin and lung, about 75% of patients bear loss of function mutations in Notch1 or Notch2 [54]. In fact, Notch upregulates p21/Cip1/WAF expression in keratinocytes, which supports its tissue-specific tumor-suppressive function in the skin [55]. A similar pattern of Notch1/2/3 functional inactivation has been reported in about 40% of bladder cancers [56]. In pancreatic adenocarcinoma, acute myeloid leukemia, and angiosarcomas, the role of Notch1 remains controversial [52,57]. 

The functional role of the other family members, Notch2, Notch3, and Notch4 is less understood. For instance, Ortica et al. had comparatively tested the function of the four Notch receptors in mouse embryonic cells by over-expressing constitutively active forms, and verified their diversity in controlling cell proliferation and differentiation fate [58]. Notably, in colorectal cancer Notch2 levels are decreased and similar results were obtained in thyroid and ovarian cancers, suggesting a tumor suppressor function for this family member [59,60]. On the other hand, Notch3 is constitutively activated in one third of basal-like breast cancers, and a Notch3 specific antagonistic antibody, anti-N3.A4, hampered the growth of orthotopic HCC1143 breast cancer xenografts in mice [61]. In prostate cancer, Notch3 overexpression was associated with higher Gleason score and a high proliferative gene expression signature [62]. In hepatocellular cancer, both Notch1 and Notch3 levels correlated with tumor grade, invasion, and metastasis [63]. In oral squamous cell carcinomas, Notch3 signaling is activated in stromal fibroblasts, in turn eliciting tumor angiogenesis [64]. Besides *NOTCH1*, also *NOTCH3* gene was found to be activated by mutations in T-ALL [65]; moreover, expression analysis of T-ALL cells revealed a common oncogenomic program triggered by both activated Notch oncogenes via RBPjk [65,66]. In other studies, Notch3 was instead linked to tumor suppressive activity, e.g., its overexpression in breast and melanoma cell lines was found to increase p21 and thereby inhibit cell proliferation and induce cell senescence [67]. Zhang et al. recently reported that Notch3 induces the tumor suppressor WWC1/Kibra, a regulator of the Hippo pathway, thus inhibiting EMT in breast cancer cells [68]. Recent data seem to support a pro-tumorigenic function of Notch4. For example, in triple negative breast cancer (TNBC) cell line MDA-MB-231, Notch4 overexpression induces proliferation and invasion and, conversely, its downregulation inhibits proliferation [69]. In another model of breast cancer—MCF7 cells—increased expression of Notch4 elicited EMT and invasiveness [70]. Notch4 is also upregulated in pancreatic cancer cell lines compared to non-transformed cells and its inhibition impairs viability, migration, and invasion [71]. Notch4 activity was also associated with EMT gene expression signature in melanoma cells and held responsible for increased metastasis [72].

An additional layer of complexity in the role of Notch signaling in cancer is added by the recent findings that the expression of several Notch target genes remains upregulated even after RBPjk depletion due to epigenetic changes (enrichment of H3K4me3 and H4ac marking the active promoters) [73]. Notably, there is controversy about the role of RBPjk in cancer: in fact, consistent with its transcriptional repressor function, RBPjk depletion can promote tumorigenesis [73,74]; however, RBPjk was also found highly expressed in glioblastomas, and its targeting decreased self-renewal of brain tumor-initiating cell and tumor formation [75]. 

## 3. Advances in Targeting of Notch Signaling by Small Molecule Inhibitors

**Figure 1:**
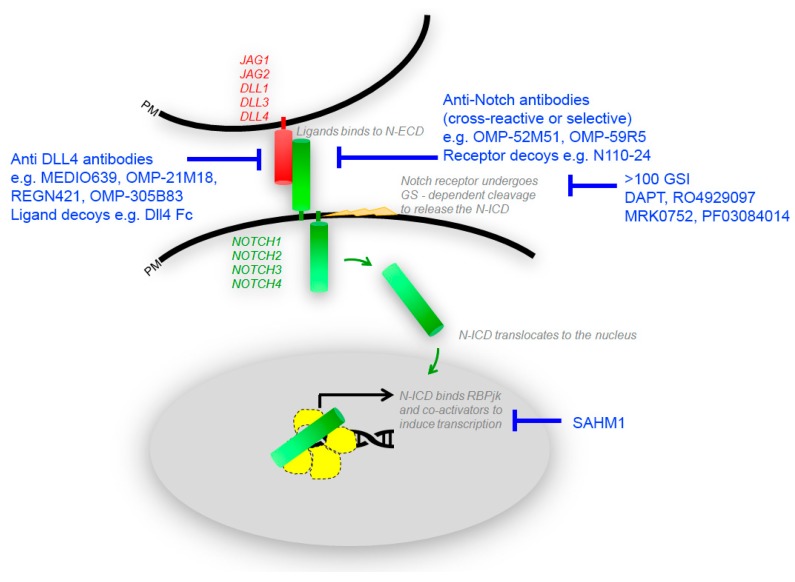
Therapeutic targets in the Notch signaling pathway. The Notch pathway can be targeted by small molecule Gamma Secretase Inhibitors (GSI), antibodies (Anti-Notch, Anti-Dll4), decoys, and peptides.

**Figure 2:**
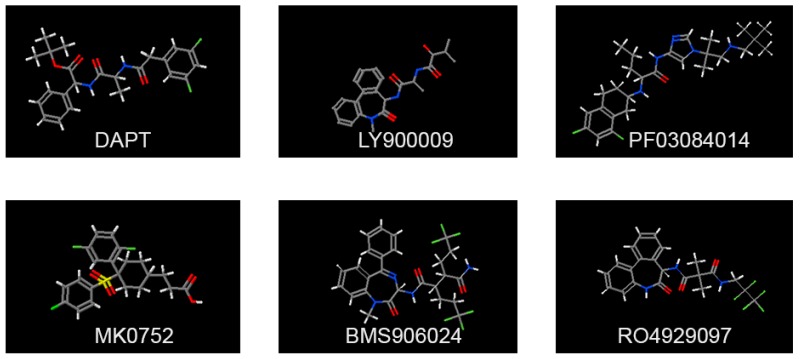
GSI structures. Structures of some commonly used GSI, which are used in vitro and in clinical trials.

**Figure 3:**
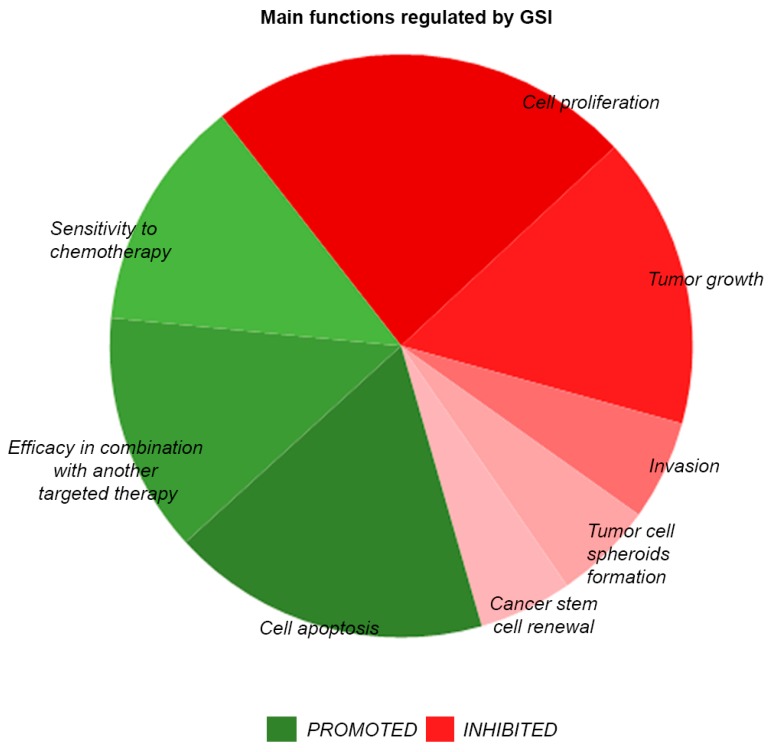
Functional effects of gamma secretase inhibitors. The pie chart represents the main biological activities promoted or inhibited by GSI, based on the prevalence of their report in literature (approx. 450 Pubmed-indexed research papers related to the use of GSI were individually analyzed).

**Figure 4:**
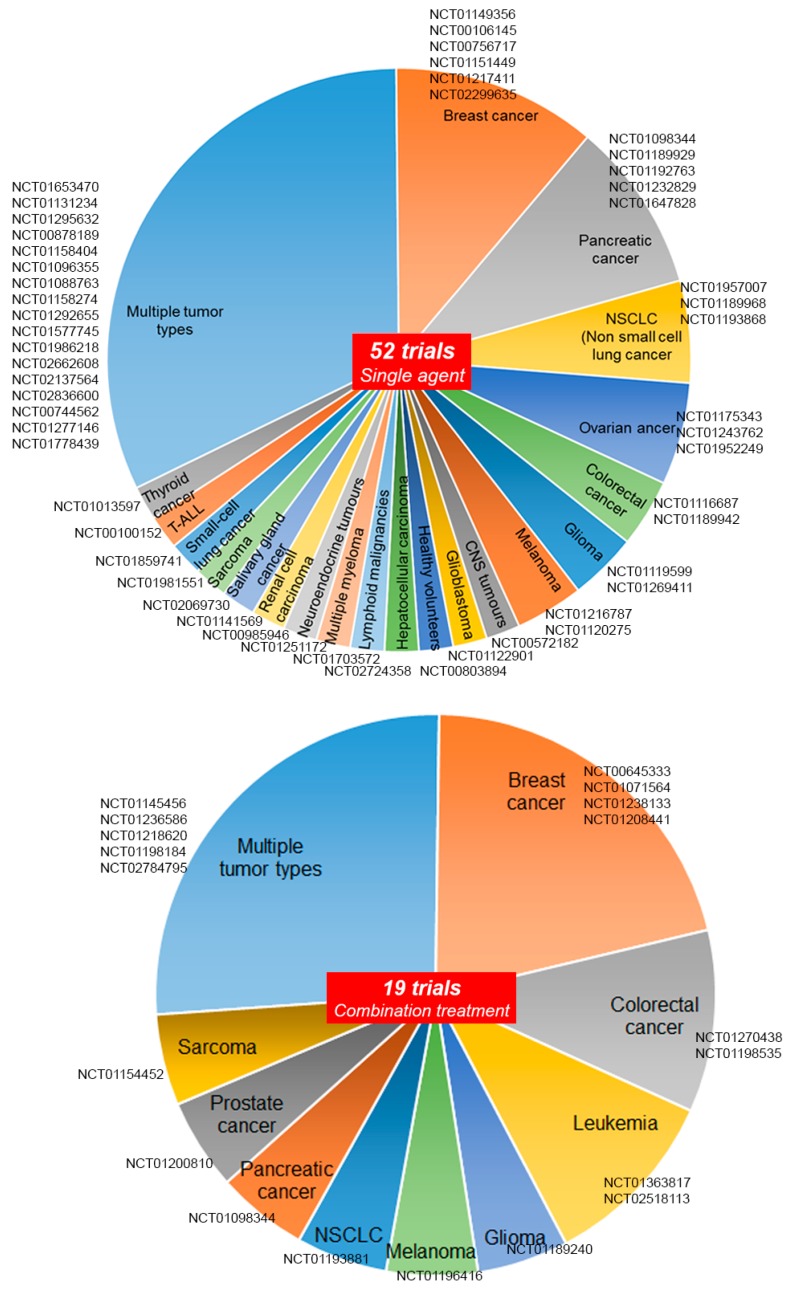
Clinical trials (named by the NCT ClinicalTrials.gov identifier) for the treatment of the indicated tumor types based on the use of Notch inhibitors, either as a single agents (upper panel) or in combination with other drugs (lower panel).

The development of small molecules able to target signaling molecules, specifically active in tumor cells, has exponentially increased over the last decades. It is relatively easier to develop targeted drugs against catalytic sites, such as for oncogenic protein kinases. Instead, the identification of appropriate targeting strategies for non-enzymatic molecules is more challenging. Based on the structure of Notch receptors and their mechanism of activation, several types of inhibitors have been generated, which are able to inhibit either the binding of the ligands or the gamma secretase-dependent proteolytic cleavage of the receptor [76] (Figure 1). These inhibitors have been tested either alone or in combination, and some of them entered clinical trials with alternating success. Gamma secretase inhibitors (GSI), which were initially developed to prevent the formation of amyloid deposits in Alzheimer’s disease, have been repurposed to block other targets of the same enzymes, including the Notch family receptors that, as mentioned, are often highly expressed in cancer. Gamma secretase is in fact a complex of proteins, including APH1, PEN2, Nicastrin, and presenilin. It has more than 100 type-I membrane protein targets, including Notch ligands, ErbB4, Syndecan, and CD44 [77,78,79]. The gamma secretase complex cleaves within the intramembrane region of its protein substrates and is very promiscuous regarding the target sequence. More than 100 GSI have been synthesized so far, with different affinity, specificity, and IC50 values, falling into 3 categories: (i) peptide isosteres, (ii) azepines, and (iii) sulfonamides [80]. GSI are either transition state analogs of the aspartyl proteinase active site (namely, they bind competitively to the catalytic site of presenilins) or non-transition state inhibitors, which bind in a site other than the active site (i.e., at the dimerization interface of the gamma secretase complex). An early generation non-transition state analogue is DAPT (*N*-[*N*-(3,5-Difluorophenacetyl-l-alanyl)]-*S*-phenylglycine t-Butyl Ester) [81]. Later, other compounds were generated, with 100-fold stronger activity, such as LY411575, LY450139, RO4929097, and BMS906024 (Figure 2). RO4929097 has been shown to extend survival in an intracranial mouse glioma model [82], whereas BMS906024 is the only GSI able to efficiently inhibit all four Notch receptors, as well as APP [83]. The non-transition state analogs, like DAPT, are supposed to be more effective than the transition state inhibitors, as substrate docking into the enzyme interior can hinder inhibitor binding in the transition state. The overall efficacy of GSIs in cancer is still under debate, as several studies have been performed in diverse models, which makes it difficult to draw univocal conclusions (Figure 3). MCF7 breast cancer cells stably expressing N1-ICD underwent EMT and grew faster in a xenograft model and, conversely, treatment of SKBR3 xenografts with DAPT modestly reduced tumor growth [84,85]. The blockade of Notch pathway by GSI-18 in mouse GBM xenografts depleted CD133-positive stem-like cells, reduced tumor growth, and significantly promoted survival [86]. These and other studies paved the way for clinical trials, in which many GSIs have been administered orally (Figure 4). The current clinical trials are focused on RO4929097, MK0752, and PF03084014. In particular, RO4929097 has been tested in phase I and II trials in patients with advanced solid tumors. In patients with colorectal carcinoma it did not show any positive effects, but instead mild toxicity with nausea and vomiting [87]. An additional, frequent adverse effect of GSI is diarrhea, associated with excess secretory goblet cells in the intestine, possibly because Notch inhibition causes an imbalance in cell fate differentiation [88]. Due to the lack of clinical benefit in multiple studies, its clinical use was terminated prematurely [89]. MK0752 has also been tested in phase I and II clinical trials, showing partial clinical benefit in a cohort of 103 patients with advanced solid tumors; one patient with anaplastic astrocytoma showed complete response lasting for more than one year [90]. Another shortcoming of GSI is their lack of effectiveness upon the onset of drug-resistance mechanisms. This has been primarily reported in T-ALL, which showed lack of benefit from GSI despite the constitutive Notch activity. GSI-resistant T-ALL cells were found to harbor mutational loss of PTEN, and thereby aberrant activation of the Akt pathway, increased glycolysis, and carbon metabolism [43]. T-ALL cell lines were also found to harbor FBW7 mutations leading to residual Notch signaling, which contributed to GSI resistance [91]. Furthermore, GSI-resistant T-ALL cells can maintain upregulated Myc expression independently of Notch, due to Brd4 activity [92].

As mentioned before, a major limitation of molecules targeting multiple Notch receptors stems from the different functions exerted by the different Notch members. They can all act as either oncogenes or tumor suppressor genes, depending on the context. Indeed, in some instances, GSI therapy was found to aggravate skin cancer [93,94]. It has also been reported that chronic treatment with Notch inhibitors leads to the formation of vascular tumors [95], consistent with the fact that Notch regulates angiogenesis, and its inhibition leads to exuberant vessel sprouting and defective maturation. Notably, only a few studies have investigated the long-term consequences of Notch inhibition. In transgenic mice undergoing progressive loss of Notch1 with age, a shorter life-span of about 10 months was observed due to widespread vascular tumors [96], such as liver hemangiomas, due to excessive proliferation of endothelial cells. In mice, GSI therapy was associated with immunosuppression and inhibition of stem cell renewal in normal tissues [97]. Intermittent regimens of systemic Notch inhibition, i.e., multiple drug cycles separated by a drug holiday, are currently being tested in an attempt to increase efficacy and minimize toxicity of GSIs. Specific targeting of the Notch pathway is also potentially achievable by tackling the N-ICD/RBPjk/MAML ternary complex (NTC) at nuclear level. For example, by using an innovative combination of molecular docking of small molecules with NTC and proximity-based AlphaScreen technology, Astudillo et al. designed a small molecule inhibitor of NTC, called Inhibitor of Mastermind recruitment-1 (IMR-1) [98]. IMR-1 selectively inhibited Notch-dependent transcriptional activation by interfering with Maml1 recruitment to the N-ICD/RBPjk complex, and impaired tumor growth in patient-derived xenograft (PDX) models. 

## 4. Combinatorial Treatments with Notch Inhibitors

Due to the lack of success of GSIs as single agents, there is now growing interest on their use in association with standard cancer treatments, including hormonal, radiation, chemotherapy, or targeted inhibitors (see Figure 1). Interestingly, cancer treatment with a range of chemotherapeutic agents, such as paclitaxel, docetaxel, cisplatin, oxaliplatin, doxorubicin, and gemcitabine independently resulted in the upregulation of the Notch pathway [99]. This clearly supports the rationale for combination therapies, since the Notch pathway may represent a survival mechanism activated to endure drug treatment. For instance, in head and neck squamous cell carcinoma (HNSCC), cisplatin-resistant tumors showed high expression of Notch1 and a good response to GSIs [100]. Similarly, in colorectal and ovarian cancers, the combination of cisplatin and GSIs was more effective than either monotherapy. In colon cancer, 5-FU and oxaliplatin induced Notch activity, which was curbed by GSI association [101]. In pancreatic cancer, in which gemcitabine chemotherapy is a standard treatment, drug-resistant tumors upregulated Notch2, Notch3, and Jag1, while the inhibition of Notch3 and gemcitabine induced apoptosis [102]. Notch pathway inhibitors have been shown to synergize with DNA damaging agents like doxorubicin in a variety of breast cancer cell lines [103,104]. TNBC is a good example of the use of combination therapy. TNBC and basal-like breast cancers often express particularly high levels of both EGFR and Notch. Dong and coworkers showed that the combined blockage of both pathways by Gefitinib and Compound-E is highly effective in suppressing HCC1806 tumor growth in a xenograft mouse model [105]. Moreover, Pandya et al. showed that a combination therapy with anti-Her2 and GSI prevents drug-resistance and tumor recurrence of HER2-overexpressing xenografts [106]. In prostate cancer, in which anti-androgen or chemotherapy is often the standard treatment, combination of docetaxel and the Notch inhibitor PF03084014 was effective in preclinical models, resulting in reduced cancer cell EMT, prosta-spheres formation, and tumor microvessel density in vivo [107]. Jin and coworkers showed a synergistic effect of the Notch inhibitor MRK003 with the Akt inhibitor MK2206, mainly impacting on invasion rather than on cancer cell proliferation [108]. In addition, in KRAS-driven lung adenocarcinomas, the co-inhibition of DDR1 (by dasatinib) and Notch pathway (by demcizumab) curbed tumor cell proliferation with additive therapeutic benefit [109]. Synergy of GSIs with the inhibition of Bcl-2 and Bcl-xL by ABT737 was also reported in multiple myeloma, in which the combined therapy resulted in the increased activity of Bak, Bax, and release of cytochrome-c, with no effects on peripheral blood mononuclear cells [110]. In T-ALL, in which Notch itself is activated in about 50% of cases, there is a population of cells that is GSI-tolerant and expands even in the presence of Notch1 inhibitors. Interestingly, increased expression of Myc in more than 50% T-ALL is reportedly due to Notch activation [26,111]. In fact, the association of GSI with a BET-bromodomain inhibitor (JQ1) downregulating Myc and Myc target genes showed increased efficacy in inducing apoptosis in T-ALL [107].

## 5. Inhibition of Notch Signaling by Blocking Antibodies and Decoys

As discussed above, GSIs inhibit not only Notch receptor activation but also other gamma secretase targets, which often lead to adverse effects and ambiguous conclusions regarding safety and efficacy. The development of therapeutic antibodies, capable of specifically inhibiting Notch family members, stands as a promising strategy to overcome this limitation. Blocking antibodies to Notch can be divided into two groups: (a) those directed to the ‘negative regulatory region’ (NRR) that enable ADAM mediated cleavage, e.g., raised against Notch1–3 by phage display technology [112]; and (b) antibodies that block receptor-ligand interaction by hindering EGF repeats. Humanized antibodies against Notch1 or Notch2/3 have been generated and entered phase I and II trials. Moreover, a cross-reactive human monoclonal Notch2 and Notch3 antagonist, OMP-59R5 (Tarextumab), is effective in reducing cancer cell proliferation and the growth of breast, lung, ovarian, and pancreatic cancers [113]. OMP-59R5 was also found to be well tolerated in advanced pancreatic cancer patients and showed efficacy in combination with gemcitabine and Nab-paclitaxel [113,114]. Notably, the use of antibodies selectively targeting Notch1 and sparing Notch2 has been shown to prevent major adverse and toxic effects related with pan-Notch inhibition [115]. Notch ligands can also be targeted by blocking molecules. For instance, anti-Dll4 antibodies have been tested in pre-clinical and clinical trials. The humanized anti-Dll4 mAb OMP21M18 caused inhibition of tumor growth in PDX models and is currently being tested in several clinical trials, either as a single agent or in combination with other drugs [116]. A neutralizing anti-DLL4 humanized phage antibody, YW152F, was shown to both specifically block Notch-DLL4 signaling and hamper the growth of MDA-MB-435 cells, implanted in the mouse mammary fat pad [117]. Combination of DLL4-blockade by either Dll4-Fc or anti-DLL4 antibodies (REGN1035, REGN421) with VEGF-trap Aflibercept showed stronger efficacy in reducing tumor burden compared to monotherapy, by blocking angiogenesis and eliciting cancer cell apoptosis in murine tumors [118]. Soluble decoys blocking Notch signaling have also been tested. For instance, Funahashi et al. generated a soluble form of the Notch1 ectodomain, which competes with Dll1 and Jag1 binding to the receptor. This molecule was particularly effective in inhibiting VEGF-induced angiogenesis in normal skin and neo-angiogenesis in tumor models [119]. Kuramoto and coworkers generated a soluble form of Dll4, called Dll4-Fc, which suppressed liver metastasis of small cell lung cancer (SCLC) cells expressing high levels of Dll4 [120]. Intriguingly, the non-canonical Notch ligand DLK1 could also compete with canonical ligands of the DSL type to block the canonical signaling pathway [121]. The Notch receptor is a membrane-tethered protein, and physiologically no soluble forms have been reported to compete with its activity. The extracellular EGF-like11-13 domains in the Notch receptor have been artificially fused to the IgG1 Fc domain to form a soluble protein capable to trap Notch ligands. Using this approach, Klose et al. showed that Notch1-EGF11-13-Fc, which showed preferential binding to Jag1, inhibited in vitro tube formation and tip cell formation in the retinal angiogenesis assay [122]. Recently, Kangsamaskin et al. generated Notch decoys specific for the unique region of ligand-receptor interaction [123]. They showed that the N110-24 decoy effectively inhibited angiogenic sprouting in the mouse retina, as well as tumor growth, by specifically blocking Jag1/Jag2 mediated Notch signaling. In contrast, the N11-13 decoy led to vessel hypersprouting in the same models by interfering with Dll1/Dll4 mediated Notch signaling. An additional approach, less explored so far, is to prevent the assembly of Notch transcription complex, composed of N-ICD, RBPjk, and MAML. For instance, Mollering et al. generated 16-amino acid long peptides, based on MAML sequence motif required for interaction with N-ICD and RBPjk, called ’stapled α-helical peptides derived from MAML1′ (SAHM). Indeed, SAHM1 prevented the assembly of active transcription complex by binding to N-ICD and RBPjk, thereby inhibiting the recruitment of the MAML1 co-activator [124]. SAHM1 peptide treatment suppressed Notch target genes in the T-ALL cell line KOPT-K1 and concomitantly halted proliferation and leukemia progression. Clearly, molecules interfering with Notch-activated transcriptional complex may be expected to recapitulate pan-Notch signaling inhibition, including some of the reported adverse effects. Moreover, the therapeutic use of peptides in human patients poses pharmacokinetic issues to be addressed.

## 6. Recent Trends in Notch Targeting by Oligonucleotide-Based Methods

Antisense oligonucleotides are short stretches of chemically synthesized DNA or RNA able to target genes of interests [125]. Due to high sequence specificity, they are expected to show less off-target effects compared to other therapeutic approaches. As nucleic acids do not easily enter inside the cells, they are generally chemically modified to increase their capacity to penetrate cell membranes. Nakazawa et al. reported that Notch signaling regulates the abnormal proliferation and differentiation of synoviocytes in Rheumatoid Arthritis. The proliferation of synoviocytes could be blocked by both Notch1 antisense oligonucleotides and the gamma secretase inhibitor MW167 [126]. Moreover, in the SCLC cell line NCI-H82, the use of Notch1-phosphorothioate antisense oligonucleotides reduced the expression of Notch target Hes1 [127]. To modulate angiogenesis, Zimrin and coworkers generated an antisense Jagged oligomer, which potentiated FGF2-induced collagen invasion by endothelial cells [128]. Very few studies have exploited these tools in vivo in preclinical models, with one limitation being the poor efficiency of intracellular delivery upon systemic administration. Notably, the systemic expression of Notch1 antisense oligonucleotides (NAS) under mouse mammary tumor virus long terminal repeat promoter was found to significantly reduce Notch activity in tissues, but also showed several Notch-associated defects on learning and memory [129,130]. Thus, an accurate titration of the systemic delivery of NAS is warranted, with regard to their application for cancer therapy. 

## 7. Conclusions and Future Perspectives

The Notch transcriptional regulator lies at the intersection of multiple signaling pathways, which control cell stemness, differentiation, survival, proliferation, migration, and invasion during development and disease. In most instances, the inhibition of Notch signaling has been shown to block cancer cell growth and angiogenesis. In fact, the deregulated expression of Notch pathway components in human cancers supports the therapeutic use of GSIs. Recent studies tend to indicate that these drugs may be more effective in combination with chemotherapy or other targeted therapies, compared to their use as single agents. The modest clinical success reached by GSIs so far could be attributed to the broad range of gamma secretase targets, resulting in toxicity and inadequate drug efficacy, thereby warranting the development of selective inhibitors directed against specific Notch pathway components. Moreover, while multiple Notch receptors are co-expressed in tumors, they do not mediate the same effects, further challenging the therapeutic efficacy of pan-Notch inhibitors. Thus, more studies are needed to understand the specific function of the different Notch receptors in cancer, as they may form distinct transcription complexes and develop specific drugs targeted to individual family members.
